# A Case of Kanamycin Self-Injection for Penile Augmentation and a Literature Review of Penile Foreign Body Injections

**DOI:** 10.7759/cureus.73325

**Published:** 2024-11-09

**Authors:** Muhammad Salik, Shahr Yar, Nityanandan G, Ahmed K Ibrahim

**Affiliations:** 1 Urology, Aberdeen Royal Infirmary, Aberdeen, GBR; 2 Urology, Glangwili General Hospital, Carmarthen, GBR; 3 Urology, Wythenshawe Hospital, Manchester, GBR; 4 Urology, College of Health and Medical Techniques, Al-Hadba University College, Mosul, IRQ

**Keywords:** foreign body penile injection, foreign substance penile injection, injection for penile augmentation, kanamycin, kanamycin penile injection, penile injection, penile injection literature review, penile self injection

## Abstract

Injection of various substances into the penis for augmentation is an uncommon and concerning practice that can have serious consequences, such as skin necrosis, ulcers, suboptimal cosmetic results, and persistent edema, often requiring extensive surgical repair.

In this article, we present a rare case of penile self-injection with Kanamycin ointment. We describe the clinical course of the disease and the management framework that was followed. Initially managed conservatively, the patient later developed voiding difficulties and skin ulcers at the injection site. Further evaluation revealed discharging sinuses and secondary ulcers. Despite initial improvement, the wounds failed to heal adequately and required specialist referral for consideration of reconstructive surgery.

The injection of foreign substances into the penis, though attempted for sexual benefits, often results in dissatisfaction and requires surgical reconstruction. Close follow-up and regular physical examination are crucial to monitor disease progression and ensure appropriate, timely management. Awareness of the harms associated with the injection of foreign substances into the penis can help reduce this practice.

## Introduction

Self-injection of foreign substances into the penis is a topic usually discussed in case reports or limited case series. As it is not a common presentation and there is variability in the substances used in such practice, there is a lack of established management guidelines. The practice is done in pursuit of penile enhancement, and these patients generally do not get the desired results and are dissatisfied [1]. Reported cases involve the injection of substances like cod liver oil [2], vaseline [3], hyaluronic acid [4], paraffin [5], and silicone [6] into the penile shaft. This carries a significant risk of complications such as pain, swelling, tissue necrosis, and paraffinomas, and often requires extensive surgical reconstruction [3,7]. Kanamycin, a known aminoglycoside, is an infrequently used substance in such practice. There are only a few reported cases [8,9]. Here, we present a case report of Kanamycin penile injection in a middle-aged man, which resulted in poorly healing ulcers on the penile shaft and required a multidisciplinary approach to address. We discuss how the case evolved to shed light on the clinical course of the disease. We also discuss the important considerations in the management of the case and describe our management and follow-up framework.

## Case presentation

**Figure 1:**
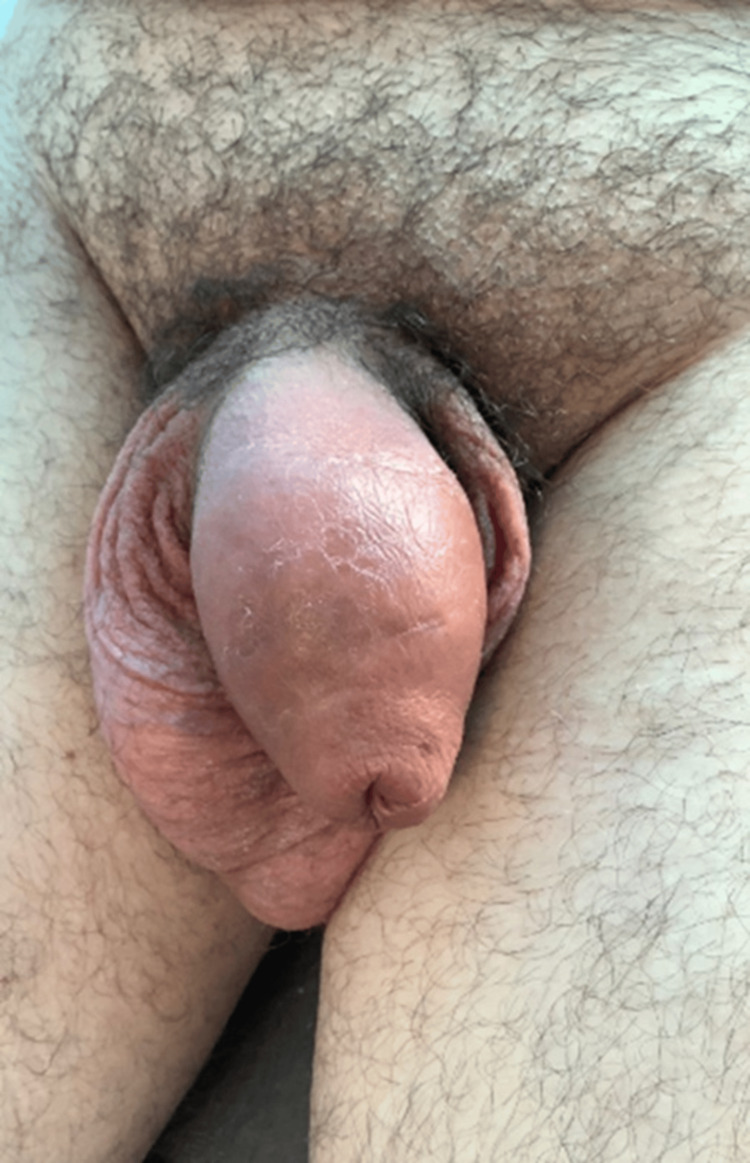
Clinical picture showing the penile swelling

A middle-aged man presented in the Surgical Assessment Unit during the COVID-19 pandemic. The patient had, on the advice of his friends, injected Kanamycin ointment into his penile shaft. The purpose of the injection was to enhance the penis and improve sexual pleasure. The patient presented to the hospital a few days after the injection with the complaint of excessive swelling of the penile shaft. There was no complaint of any urinary symptoms at this presentation. Upon examination, there was extensive circumferential swelling of the penile shaft, extending slightly to the base of the penis and the upper part of the scrotum. The swelling was hard in consistency, well-defined, and mildly tender. The rest of the scrotum was normal, with normal testicular and groin examination (Figure 1).

The patient was able to pass urine, was afebrile, and was otherwise well within himself. Therefore, the decision was made to manage this conservatively in the first instance and monitor the progression. He was discharged home after safety netting advice regarding infection and urine retention. An outpatient appointment was booked to monitor the progression of the condition.

**Figure 2:**
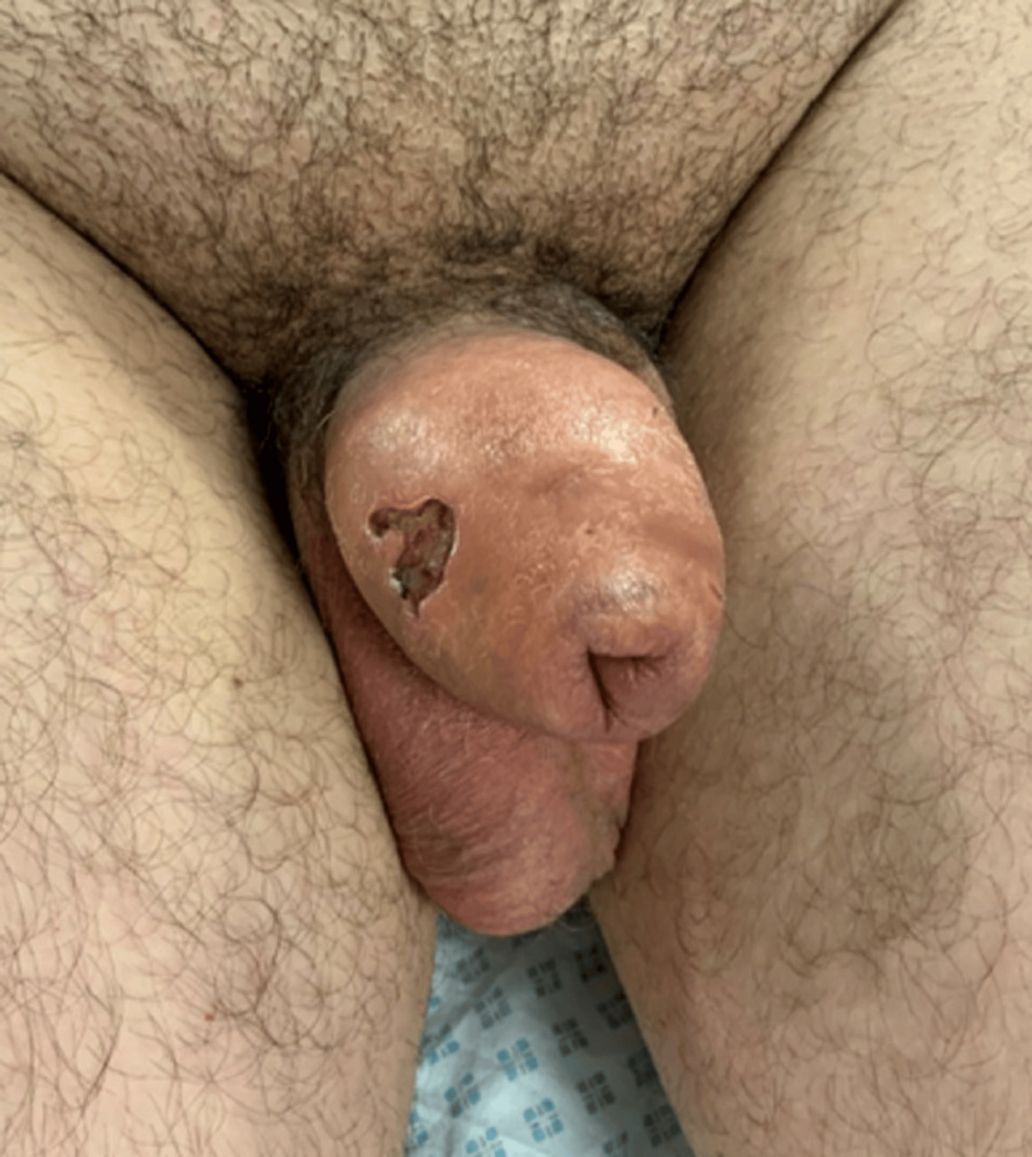
Clinical picture showing the sinuses and excoriation on the shaft of the penis

The patient was seen again in three weeks. By then, the patient had developed voiding difficulties. He also complained of discharge from the injection site. Upon examination, the swelling had increased, with an open sinus tract on the shaft of the penis, which was the initial injection site (Figure 2). According to the patient, the site discharged yellowish material similar to the original Kanamycin ointment.

**Figure 3:**
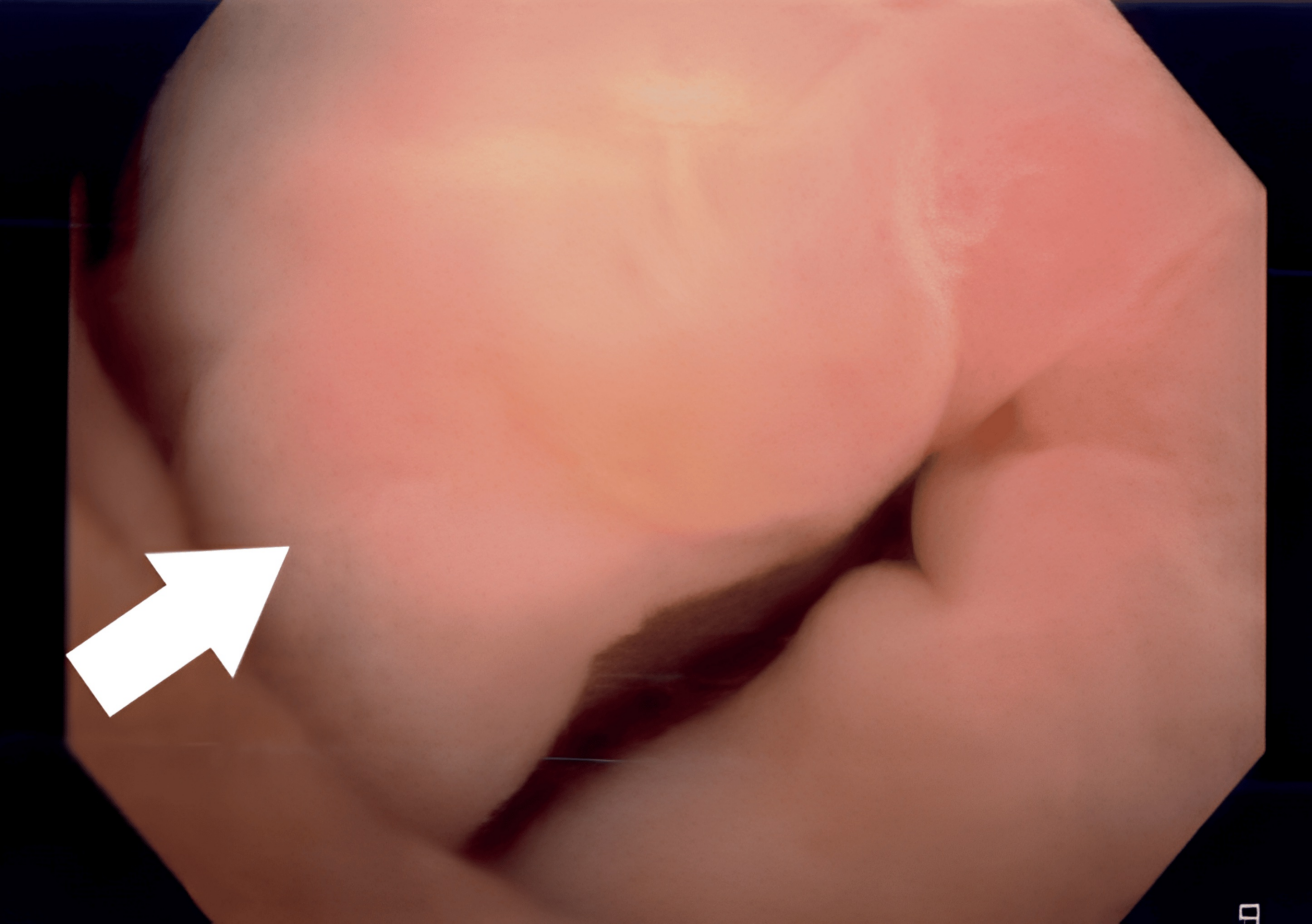
Flexible cystoscopy image showing swelling of the foreskin and the soft tissue around the meatal opening

The decision was made to catheterize the patient. The swelling had progressed further, and therefore, catheterization proved to be difficult. A flexible cystoscopy was performed, which did not reveal any concerning findings other than excessive swelling of the foreskin and the glans (Figure 3). The urethra appeared to be normal, and no abnormality was seen in the bladder. A catheter was placed over a guide wire.

**Figure 4:**
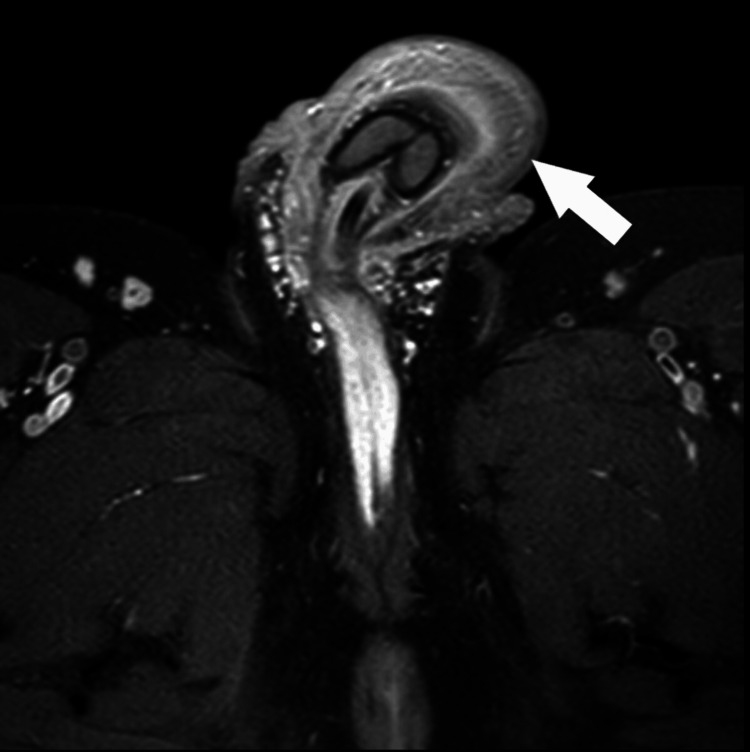
Axial plane MRI image showing soft tissue swelling

**Figure 5:**
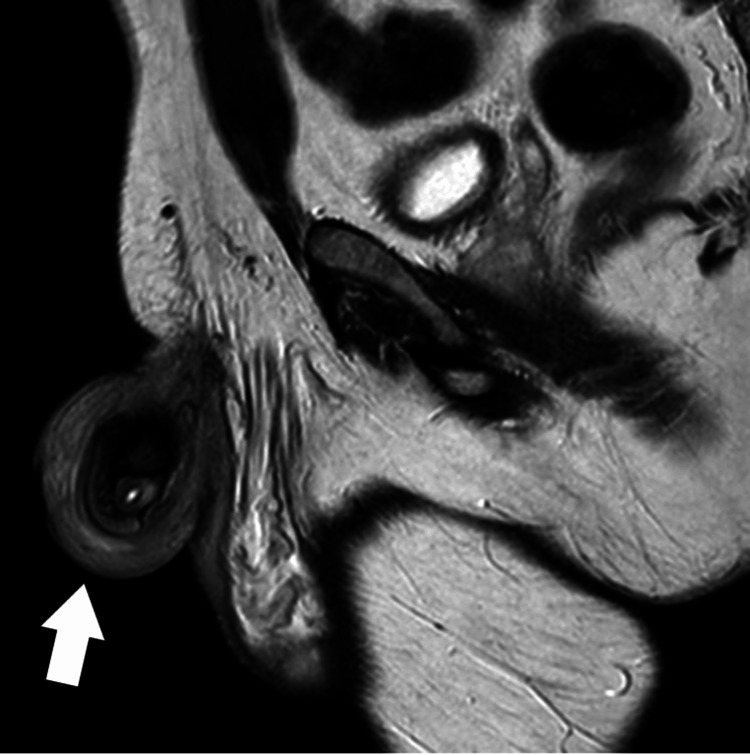
Sagittal plane MRI Image showing soft tissue swelling

An MRI scan was arranged to assess the extent and for further characterization. The MRI scan revealed no underlying damage or significant infiltration. It showed soft tissue swelling, but fortunately, normal anatomy with no fibrosis (Figures 4-5).

**Figure 6:**
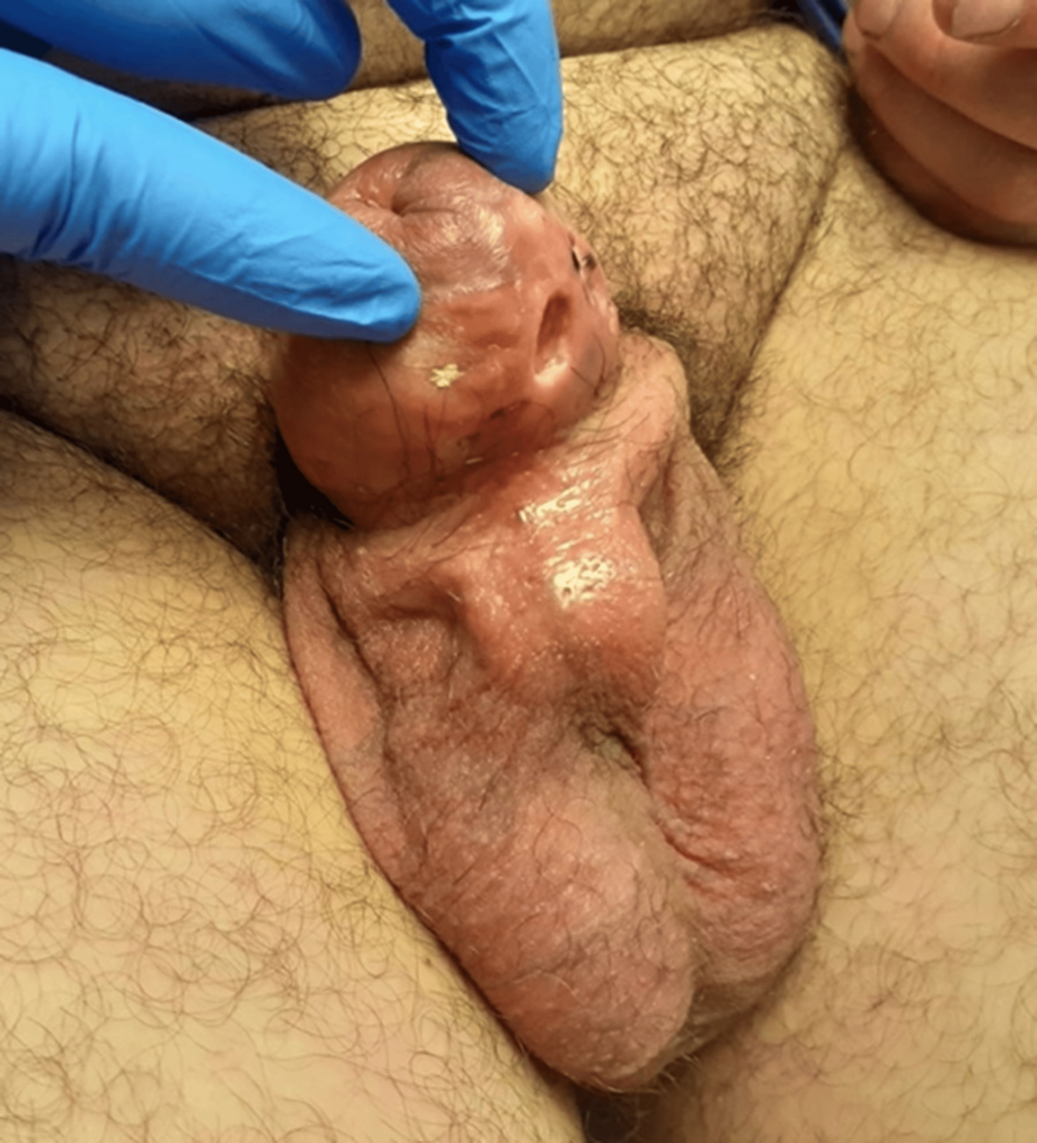
Active secondary ulcers two to three weeks after formation

The patient was seen again in four to six weeks. A trial without a catheter was attempted, and the patient was able to pass urine without a catheter. At this time, his primary ulcer had healed. However, new secondary ulcers had started forming (Figure 6), having a similar yellowish discharge as the first ulcer. This was considered a failure of conservative management, and therefore, the patient was referred to the Plastic Surgery Unit of a specialist center for further management and consideration for reconstruction.

The patient was offered plastic surgical reconstruction of the penile skin; however, he did not attend any further follow-up appointments and therefore was lost from the system. Whether he sought treatment elsewhere, or his condition self-resolved with the healing of his secondary ulcers and he did not require any further management, remains unknown.

## Discussion

The perception of having an inadequate penis and the desire to enhance sexual pleasure are considered to be the main drivers behind injecting foreign substances into the penis. This malpractice is becoming more common and is reported by people from all over the globe [10]. The most common injection site is the penile shaft, in 94.3%, and almost half of the patients have more than one injection site [10].

As per our knowledge, Kanamycin self-injection for penile augmentation has not been described before in our region, the United Kingdom. Our case report further highlights the association of Kanamycin penile injection with urinary symptoms and the temporary need for catheterization, a situation described once before in the literature in a similar context [9]. Careful clinical examination is required to assess the extent of the complications. Other diagnostic modalities, such as flexible cystoscopy and an MRI scan, may be needed for further assessment.

Some cases can be managed conservatively; however, most patients would require surgical treatment, such as wound excision and primary closure, scrotal flap, circumcision, and other reconstruction techniques [10-12]. A table, formulated after the literature review, informing different materials used in such practice, the associated complications, and the treatment modalities used, is given in Table 1.

**Table 1:** Literature review of foreign substances used in penile injections

Foreign body material used	Reference of reporting source	Reported complications	Reported treatment options utilized
Cod liver oil	Al-Ansari et al. (2010) [2]	Paraphimosis, abscess formation, necrosis of penile skin	Dorsal slit, debridement, local penile flap, and V-Y plasty
Vaseline	Nyirády et al. (2008) [3]	Pain and severe phimosis	Complete and radical excision with surgical reconstruction
Liquid paraffin/mineral oil	Hrudka et al. (2022) [5]	Recurrent paraffinoma	Subcutaneous excision with surgical reconstruction using skin graft
Baby oil	Ahmed et al. (2017) [13]	Swelling, pain, edema causing phimosis, skin necrosis	Steroids and antibiotics to limit edema and infection. Debridement of necrotic areas, circumcision, skin graft
Silicone	Ahmed et al. (2017) [13]	Recurrent intermittent inflammation of Indurated nodular masses along the shaft of the penis causing pain during intercourse	Surgical removal of silicone deposits
Mechanical oil	Ahmed et al. (2017) [13]	Significant persistent edema making penetrative intercourse difficult	Planned for excision and full-thickness grafting but the patient decided against it
Jamaica oil	Francis et al. (2014) [14]	Swelling, scar formation, painful erections, ulcer formation, fibrosis	Conservative management, surgical debridement, reconstructive surgery (scrotal flap reconstruction)
Mercury	Oh et al. (2007) [15]	Distant spread of mercury	Total phallectomy and perineal urethrostomy, followed by chelation therapy
Olive oil	Wiwanitkit (2004) [16]	Penile pain	Surgical removal of foreign body material
Kanamycin	Exterkate and van Basten (2020) [9]	Swelling, temporary problems with urination, painful erections	Surgical excision of foreign body material
Medical application in a healthcare setting			
Hyaluronic acid	Quan et al. (2021) [4]	Subcutaneous bleeding, subcutaneous nodules, swelling, and infection. The reported rate of complications rate was low at 4.3%. The injections in this study were administered by surgeons in a healthcare setting for the purpose of penile augmentation.	Conservative management, surgical debridement, surgical removal of nodules, antibiotics, hyaluronidase therapy
Polymethylmethacrylate (PMMA)	Casavantes et al. (2016) [17]	Penile injections are done by a healthcare professional for the purpose of penile augmentation. The reported rate of complications is very low, at 0.4%. The complication mentioned is PMMA nodules.	Surgical removal of PMMA nodules

Our case report highlights the protracted nature of disease progression when Kanamycin is used in penile injection and aims to provide an insight into the clinical course of the disease, i.e., initial circumferential swelling leading to delayed development of the primary ulcer and urinary symptoms, followed by healing of the primary ulcers but development of secondary ulcers.

As documented in our case report, such patients require follow-up visits to assess their progress and detect any further complications. In the current post-COVID-19 healthcare system, in which virtual clinic follow-ups are prevalent, it is important to see these patients face to face to ensure proper assessment of the genital skin and urinary symptoms. The need for a urethral catheter should be kept in mind when assessing patients in subsequent follow-ups.

**Figure 7:**
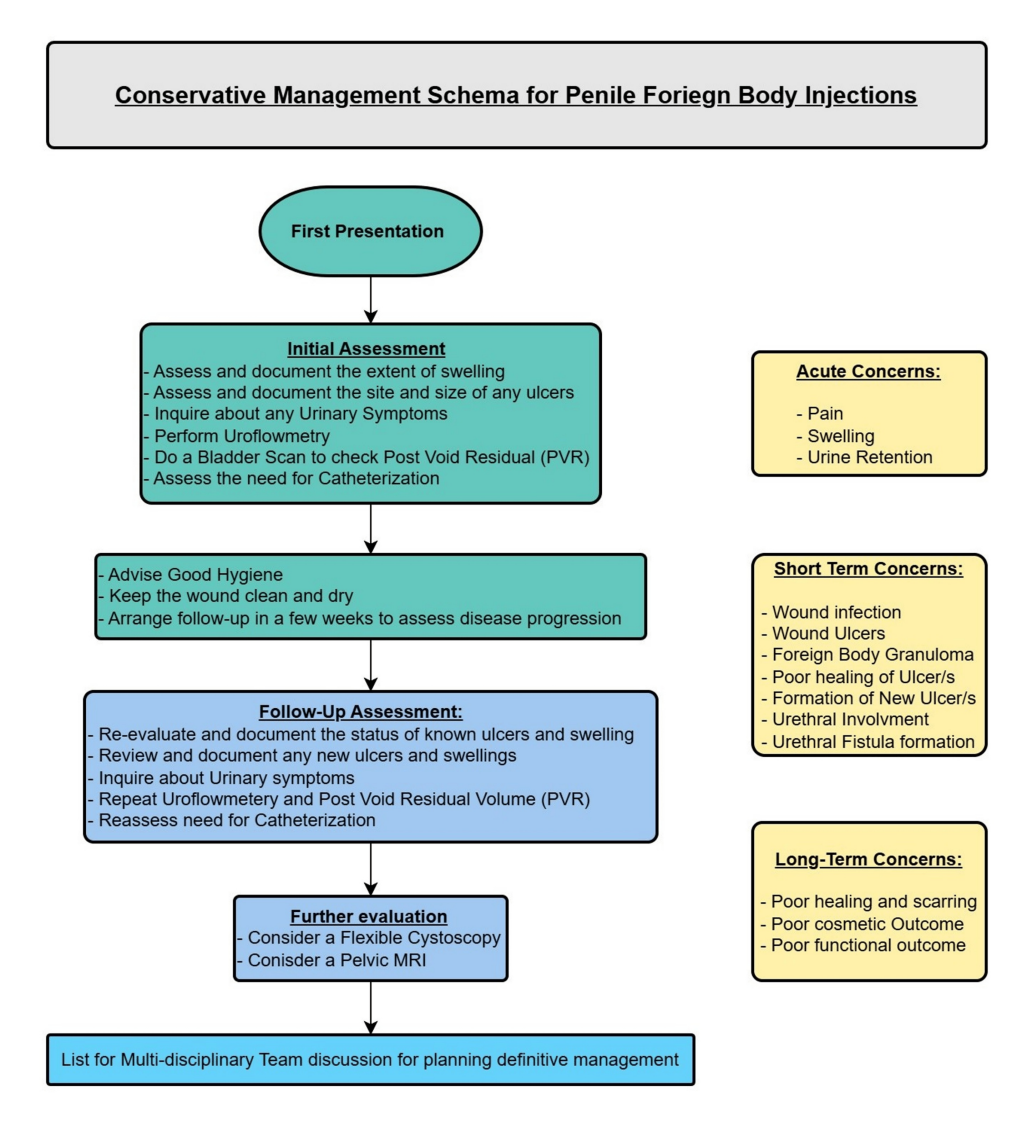
Suggested conservative management schema for penile foreign body injections

From this case report, the following suggestions can be made regarding the management of similar cases: the pertinent clinical concerns in similar cases would be urethral inflammation and resultant urinary retention in the acute setting. Doing a bladder scan would be advisable for documentation and baseline reference at presentation. Long-term concerns include infection, poor and delayed healing of the ulcers, formation of new ulcers, poor cosmesis, and sexual dysfunction. Hygiene advice, including daily change of undergarments, routine washing of the genital area with clear water to keep the wound clean, and advice to dry the area appropriately to avoid infections, would be helpful. An MRI scan can be considered to allow the characterization of any residual foreign material within the layers of the penile architecture and to assist in further management planning. A flexible cystoscopy can be helpful in assessing the integrity and patency of the urethra. Figure 7 offers a flow chart of the conservative management pathway that we followed in this case.

## Conclusions

Injection of foreign substances into the penis is an uncommon presentation, and Kanamycin is a rare substance to be used in such practice. General principles of management of penile self-injection should be kept in mind when dealing with patients who may present with an injection of a rare or unknown substance into the penis. Kanamycin penile injection cases can present with swelling and progress to form primary and secondary ulcers. Conservative management can be considered, depending on the presentation. Further case reports are required to assess the longer-term consequences of Kanamycin penile injection and to ascertain if the disease is self-limiting or requires surgical intervention. Given the preventable nature of the disease, it is important to spread awareness regarding the harms and complications of injecting foreign substances into the penis.

## References

[1] Moon DG, Yoo JW, Bae JH, Han CS, Kim YK, Kim JJ (2003). Sexual function and psychological characteristics of penile paraffinoma. Asian J Androl.

[2] Al-Ansari AA, Shamsodini A, Talib RA, Gul T, Shokeir AA (2010). Subcutaneous cod liver oil injection for penile augmentation: review of literature and report of eight cases. Urology.

[3] Nyirády P, Kelemen Z, Kiss A, Bánfi G, Borka K, Romics I (2008). Treatment and outcome of vaseline-induced sclerosing lipogranuloma of the penis. Urology.

[4] Quan Y, Gao ZR, Dai X (2021). Complications and management of penile augmentation with hyaluronic acid injection. Asian J Androl.

[5] Hrudka J, Gregušová A, Čapka D (2022). Penile paraffinoma: a case report. Cesk Patol.

[6] Plaza T, Lautenschlager S (2010). Penis swelling due to foreign body reaction after injection of silicone. J Dtsch Dermatol Ges.

[7] Jeong JH, Shin HJ, Woo SH, Seul JH (1996). A new repair technique for penile paraffinoma: bilateral scrotal flaps. Ann Plast Surg.

[8] Oanta A, Irimie M, Rogoz S, Oanta S, Lupu S (2013). Penile paraffinomas after self-injection with kanamycin ointment. Bull Transilv Univ Brasov Med Sci.

[9] Exterkate L, van Basten JPA (2020). Case report. Paraffinoma of the penis after injection with kanamycin ointment (Article in Dutch). Tijdschrift voor Urologie.

[10] Pang KH, Randhawa K, Tang S (2024). Complications and outcomes following injection of foreign material into the male external genitalia for augmentation: a single-centre experience and systematic review. Int J Impot Res.

[11] Faveret PL, Santiago F (2018). Surgical management of penile lesions secondary to foreign body reaction: a case report and systematic review. Aesthet Surg J.

[12] Svensøy JN, Travers V, Osther PJ (2018). Complications of penile self-injections: investigation of 680 patients with complications following penile self-injections with mineral oil. World J Urol.

[13] Ahmed U, Freeman A, Kirkham A, Ralph DJ, Minhas S, Muneer A (2017). Self injection of foreign materials into the penis. Ann R Coll Surg Engl.

[14] Francis J, Poh Choo Choo A, Wansaicheong Khin-Lin G (2014). Ultrasound and MRI features of penile augmentation by "Jamaica Oil" injection. A case series. Med Ultrason.

[15] Oh KJ, Park K, Kang TW, Kwon DD, Ryu SB (2007). Subcutaneous metallic mercury injection for penile augmentation. Urology.

[16] Wiwanitkit V (2004). Penile injection of foreign bodies in eight Thai patients. Sex Transm Infect.

[17] Casavantes L, Lemperle G, Morales P (2016). Penile girth enhancement with polymethylmethacrylate-based soft tissue fillers. J Sex Med.

